# Perceived Parenting Mediates Serotonin Transporter Gene (5-HTTLPR) and Neural System Function during Facial Recognition: A Pilot Study

**DOI:** 10.1371/journal.pone.0134685

**Published:** 2015-09-29

**Authors:** Saori Nishikawa, Tamotsu Toshima, Masao Kobayashi

**Affiliations:** 1 Department of Pediatrics, Graduate School of Biomedical and Health Sciences, Hiroshima University, Hiroshima City, Hiroshima, Japan; 2 Department of Psychology, Graduate School of Education, Hiroshima University, Higashi Hiroshima City, Hiroshima, Japan; 3 Phoenix Leader Education Program, Hiroshima University, Hiroshima City, Hiroshima, Japan; University of California, San Francisco, UNITED STATES

## Abstract

This study examined changes in prefrontal oxy-Hb levels measured by NIRS (Near-Infrared Spectroscopy) during a facial-emotion recognition task in healthy adults, testing a mediational/moderational model of these variables. Fifty-three healthy adults (male = 35, female = 18) aged between 22 to 37 years old (mean age = 24.05 years old) provided saliva samples, completed a EMBU questionnaire (Swedish acronym for Egna Minnen Beträffande Uppfostran [My memories of upbringing]), and participated in a facial-emotion recognition task during NIRS recording. There was a main effect of maternal rejection on RoxH (right frontal activation during an ambiguous task), and a gene × environment (G×E) interaction on RoxH, suggesting that individuals who carry the SL or LL genotype and who endorse greater perceived maternal rejection show less right frontal activation than SL/LL carriers with lower perceived maternal rejection. Finally, perceived parenting style played a mediating role in right frontal activation via the 5-HTTLPR genotype. Early-perceived parenting might influence neural activity in an uncertain situation i.e. rating ambiguous faces among individuals with certain genotypes. This preliminary study makes a small contribution to the mapping of an influence of gene and behaviour on the neural system. More such attempts should be made in order to clarify the links.

## Introduction

Recognition of facial expressions influences our emotion and social behaviours. Autism spectrum disorder (ASD) is characterized by deficits in communication and social interactions [1], and autistic-like behaviors are seen even among healthy individuals [2, 3]. In neuroimaging investigations, associations were found between autistic traits and frontal activation when participants were required to recognize negative expressions [4]. In contrast, an NIRS (Near-Infrared Spectroscopy) study showed that participants with PDD (Pervasive Developmental Disorder) showed reduced oxy-Hb changes during presentation of scared faces [5]. Cognitive rehabilitation has a positive influence on chronic prefrontal lesion patients’ prefrontal activity during a facial recognition task [6].

Over the past few years there have been an increasing number of studies using an endophenotypic approach, which serve to bridge biology and psychology. The serotonin transporter gene (5-HTTLPR) is one of the most frequently studied genes relating to personality and behaviour. The 5-HTTLPR polymorphism is based in the promoter region of the gene, with two variants commonly distinguished- the long or L-allele and the short or S-allele. Early studies indicated that SS individuals show an increased risk of depression [7, 8], which might relate to the finding that amygdala responding to negative stimuli is greater among SS individuals [9, 10]. 5-HTTLPR has also been found to moderate an association between emotional behavior and marital satisfaction [11]. Some studies have reported influences of 5-HTTLPR on social anxiety. It was reported that coping potential appears to be reduced in SS carriers [12], who appear to be less successful in reducing negative affect via cognitive reappraisal [13]. These researchers provided support for a mediational model in which 5-HTTLPR contributed to social anxiety symptoms via decreased reappraisal. Another study indicated that negative self-reflection provokes stronger personal distress in SS than in LL individuals [14]. Compared to the LL individuals, SS individuals report higher levels of emotional reactivity (i.e. empathy, self-conscious emotions) [14], are more sensitive to negative views of the self [4], and show more personal distress and physiological arousal when looking at other people’s distress [15]. Among a sample of high-exposure 9/11 survivors, SS individuals reported more post-traumatic stress symptoms (PTSS) than SL/LL individuals, and that both PTSS and the presence of short allele predicted decreased activity in several cortical midline regions when viewing 9/11 images [16]. One study investigated genetic patterns in relation to PMO (Personal Meaning Organization). S allele carriers were more likely to develop an Outward PMO, in which individuals are vulnerable to negative social judgments and tend to perceive their early attachments as less predictable [17].

Early parent-child interactions influence the development of the amygdala and prefrontal cortex [18,19]. More specifically, there is a relationship between infant attachment quality at 18 months and prefrontal region activation during emotion regulation in adulthood [20]. Parental bonding styles during childhood have an impact on recognition of facial emotions later in adulthood. Zeng et al [21] showed an influence of early childhood experience with parents on the decoding process and sensitivity during the processing of emotional facial expression in adulthood. Autonomy from mothers predicts fear recognition accuracy, and maternal care predicted sadness recognition accuracy. The important influences of parenting behaviour and attachment style on emotional wellbeing are well-known [22], and such effects may be amplified as a function of genotype [23]. Individual differences in adapting to different environments might be illuminated by genetic variation. Thus, behaviour may depend on both genes and environment. There have been some attempts to identify links between genes and environment (G×E) or gene and gender interactions on emotional/ behavioural problems and emotional processing or regulation. It was reported that 5-HTTLPR influences impulsive behaviour via memories of paternal and maternal rearing styles among adults [23]. There are effects of 5-HTTLPR on emotion recognition, as well as a gene-environment interaction, with childhood emotional abuse as well as recent life events [24]. Owens et al. [25] supported a moderated model of the effects of 5-HTTLPR and early childhood adversity on cognitive and emotional processing in adolescence. Adolescents who carried the S-allele and who were exposed early childhood adversities were less able to classify negative and neutral stimuli compared to S allele carriers who did not experience early childhood adversities. Influences from both genes and environment show increased stability with age [26]. Gender appears to play different roles in facial recognition. For example, SS homogenous females recognized sadness or anger more quickly than females with other genotypes, whereas a male heterozygous group were quicker than their counterparts with other genotypes [27].

Above all, interpreting results from an endophenotype approach is complex. More recently, studies have suggested that the SS genotype serves as a plasticity factor [28] rather than a risk factor, as argued previously [7, 8]. The SS genotype can be associated with both negative and positive reactions [28]. It was also noted that the effects of genes and environment on outcome could depend on the methodology by which environments are assessed [29]. There have been ethnic differences reported for the association between 5HTT and depression [30], as well as brain function [31, 32]. To our knowledge, the neural influences on the genetic and environmental basis of social cognition among healthy individuals remains poorly understood, particularly for Japanese samples. Furthermore, we sought to address the question of whether perceived parenting mediates or moderates 5-HTTLPR and right/left frontal activation during a facial recognition task, as measured by NIRS. Therefore, the aim of the present study was to assess changes in prefrontal oxy-Hb levels during a face-emotion recognition task in healthy adults. The intent was to examine the interplay between serotonin transporter gene (5-HTTLPR), perceived parenting style, and their effects on cognitive/emotional processing. This investigation was a pilot study using 2ch NIRS, and we focused on the prefrontal cortex, according to earlier studies showing links between the prefrontal cortex and facial expression recognition, e.g. [6, 33]. In light of the reported links between genes, attachment with early caregivers, and behaviour, we hypothesized there would be relationships between the 5-HTTLPR genotype and neurological system functioning that are mediated or moderated by perceived parenting style. More specifically, it was expected that a higher perception of parental warmth would predict better face recognition, and this effect would depend on the individuals’ genotype.

## Materials and Methods

### Participants and Procedure

Fifty-three healthy adults (males = 35, females = 18) aged between 22 to 37 years (mean age = 24.05 years old) were recruited from a university in Hiroshima. This study was approved by the Ethics Committee of Hiroshima University, and the procedure was followed in accordance with the Declaration of Helsinki [34]. All participants were voluntary and gave their written informed consent. After a brief explanation of the present study, participants provided their informed consent, a saliva sample for DNA analysis, completed a questionnaire assessing perceived parental style, and completed NIRS recording. The entire procedure took approximately 30–40 minutes.

### Facial-emotion Recognition Task

**Fig 1:**
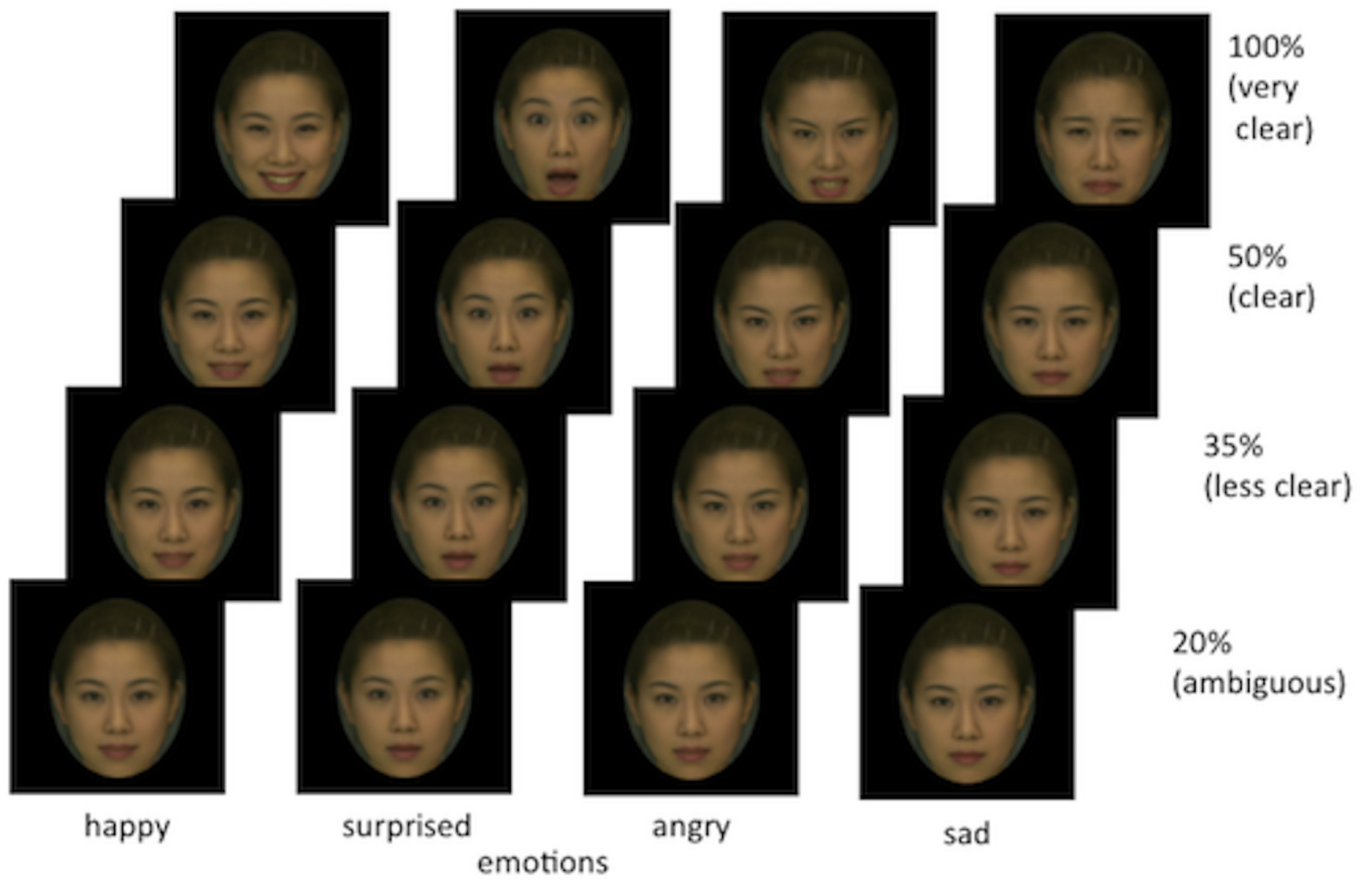
Emotional facial expressions used in the experiments and intensity of emotion from 20% (ambiguous), 35% (less clear), and 100% (clear). Note: Permission for publication of images was obtained by Hiroshi Yoshida. Publication of morphed images complies with the Terms of Use of the DB99 database.

**Fig 2:**
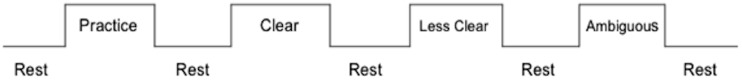
Experimental design for NIRS recordings.

**Fig 3:**
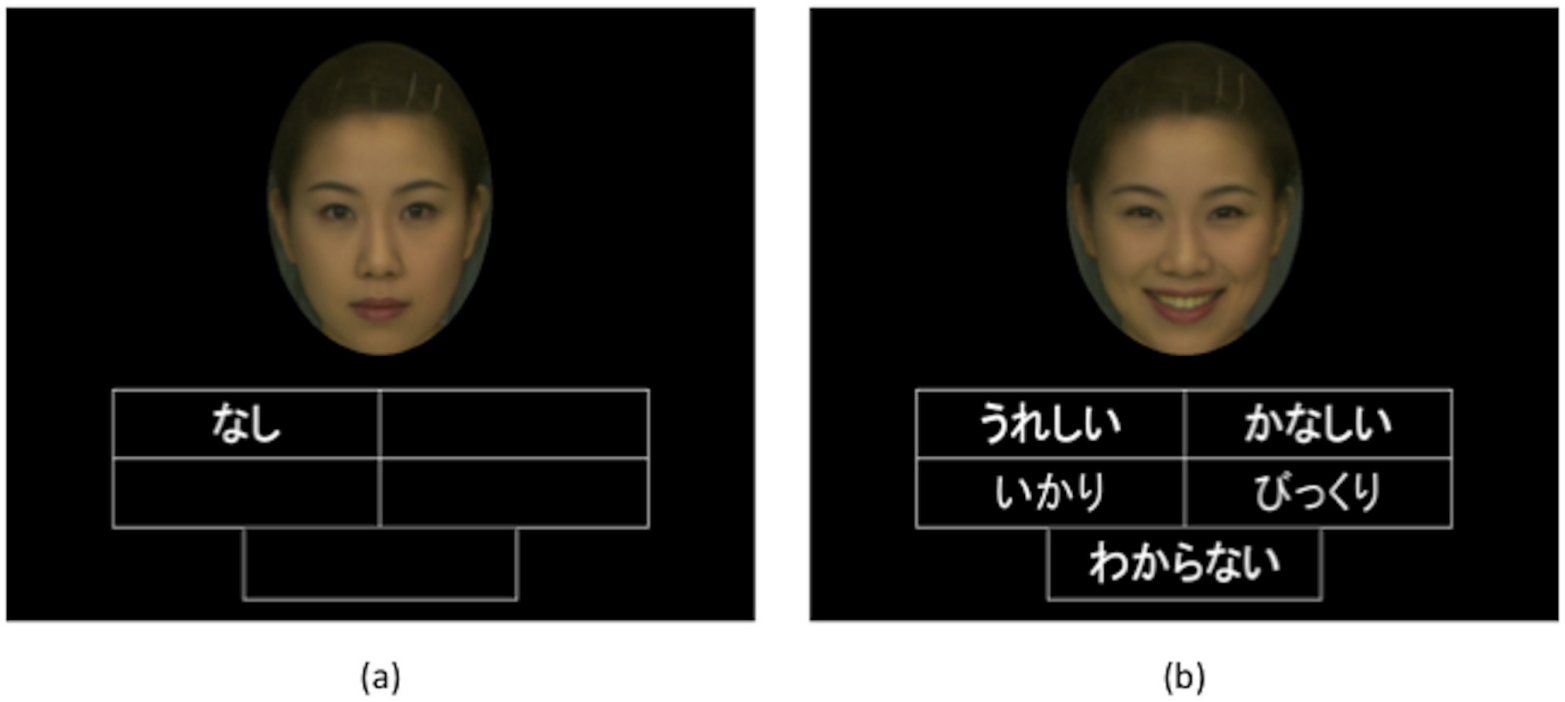
(a) & (b). Screen shots of the facial-emotion task. Note: (a) A screenshot of the control task with a neutral facial expression. In the box, the participants are asked to click on a text “None”, (b) a screenshot of the task—the participants are asked to click on the best description of the female expression in the centre of the screen. The descriptions in the boxes from left to right: “Happy”, “Sad”, “Angry”, “Surprised”, “I have no idea”. Note: Permission for publication of images was obtained by Hiroshi Yoshida. Publication of morphed images complies with the Terms of Use of the DB99 database.

Six hundred coloured images depicting four basic emotional expressions (happiness, surprise, anger, sadness) were used. We used standardized images that were developed using a morphing technique from database DB99 (Advanced Telecommunications Research Institute International, Inc, Nara, Japan, http://www.atr-p.com/products/face-db.html). These images have been used in prior studies, e.g. [6, 35]. We prepared one neutral and four emotional expressions from 50% (clear) to 35% (less clear) and 20% (ambiguous) in terms of expression clarity, by morphing photos of the women (see Fig 1). Each image was framed by an oval to avoid influences of hairstyle and clothing. One participant per session arrived at the laboratory and was seated in front of a computer with an NIRS recording device attached. The experimental setting is shown in Fig 2. The stimuli were presented in colour. One image was displayed at a time, centred on the monitor, with four expression descriptors (“happy”, “sad”, “angry” and “surprised”) displayed at the bottom (see Fig 3a & 3b). The participants were asked to click on the best description of the female expression in the centre of the screen. A neutral face without emotion was used for the baseline measurement. The participants were told to click on the text “I have no idea” if the expression was too difficult to rate. According to previous findings using the facial-emotional recognition task with both male and female faces [36], differences in the effects of male and female faces are relatively small. In order to simplify the task for the participants, we used only female faces in the present study.

### NIRS recordings

To monitor brain activation during the facial-emotion recognition task, we used a non-invasive neuroimaging technique, NIRS (Near-Infrared Spectroscopy). Using a 2-ch NIRS system (NIRO-200NX C10448), with a reflectance mode with different wavelengths (735, 810, 850 nm), hemodynamic concentrations in the cortex were measured. The NIRS probes consist of two sets of one detector and one emitter, which are attached at FP1 and FP2 respectively according to the international 10–20 EEG electrode system. Two probe holders were placed on the left and right sides of the forehead over the eyebrow with double-sided adhesive tape. In order to reduce artifacts, the participants were asked to sit on a chair and not to move any part of their body unrelated to the task. Assuming that the differential path length is 24 cm, the changes in the chromophore concentration were expressed in micromoles per litre. The time resolution of the NIRS recordings was every 1 s. Results based on changes in oxy-Hb concentration were reported, because this is the most sensitive hemodynamic response parameter. First, baseline correction was performed at the pre/stimulus period (-25-0s) for each run. The corrected data were then averaged across two runs and converted to *z*-scores (by dividing the difference between means during the pre-stimulus and stimulus (10-30sec) periods by the standard deviation during the pre stimulus period). Unlike fMRI, NIRS recordings allow subjects to keep their body positions and the experimental setting is not stressful.

### Perceived parental rearing

The EMBU (Egna Minnen Beträffande Uppfostran–“My memories of upbringing”) [37] is originally a Swedish self-report measure of adult perceptions of the rearing behaviour of one’s parents, and includes Rejection, Emotional Warmth, Overprotection, and Favouring Subject subscales. The EMBU consists of 81 items each rated on a 4-point Likert scale (1–4). A Japanese version of the EMBU has been developed and shown to have good psychometric properties [37]. According to the mean values provided by Someya et al [38], participants were divided into high/low rejection or warmth groups for some of the statistical analyses.

### Genotyping

Genomic DNA was extracted from oral mucosa collected from participants using the QIAamp DNA Micro Kit (QIAGEN, Tokyo, Japan). This study was performed using ethnically homogeneous individuals (only of Japanese descent). The serotonin transporter gene (5-HTTLPR) of the 5-HTT gene regulatory region was amplified via polymerase chain reaction (PCR) with forward (59-GGCGTTGCCGCTCTGAATGC-39) and reverse primers (59-GAGGGACTGAGCTGGACAACCAC-39). For PCR, 10 ng of genomic DNA was used in a 25-mL reaction mixture containing 0.5 U of KOD FX Neo (Toyobo Co., LTD.) and 10 pmoles of each primer in PCR buffer for KOD FX Neo (Toyobo Co., LTD.). Cycling conditions were as follows: Denaturation (94uC for 2 min) and 30 cycles of amplification (98uC for 10 sec, 63uC for 30 sec, and 68uC for 30 sec). PCR products were separated by electrophoresis in a 3% agarose gel and visualized by UV after ethidium bromide staining. A 484 bp band was observed for the S allele, and a 528 bp band for the L allele; heterozygous samples showed both alleles. Two investigators scored allele sizes independently and any inconsistencies were reviewed and rerun.

### Data Analysis

SPSS (The Statistical Package for Social Sciences) version 22 [39] was used for computing/performing descriptive statistics, correlations, and ANOVAs (analysis of variance). SEM (Structural Equation Models) were analysed using AMOS 22 on SPSS to evaluate the mediating effects of the variables specified by path diagram models. This software performs analyses of moment structures through maximum likelihood estimation. To investigate whether a variable X (PARE) is a mediator between independent variable A (5HTT) and dependent variable B (RoxH) in the path analysis, a direct path from A to B is drawn during a first analysis. In the next analysis, two paths are added, one from A to X and the other from X to B. If X is a significant mediator, the weight of the path from A to B will decrease in the second analysis in comparison to the first one [40]. The GFI (Goodness of Fit Index) is considered a reasonable statistical index for evaluating a model and assesses the fit between a hypothesized model and the data. AMOS calculates all measures that capture model evaluation that were selected based on different theoretical perspectives, including CMIN/df (the minimum value of sample discrepancy divided by its degree of freedom, smaller values preferable [41]), CFI (The Comparative Fit Index, a measure of the relative amount of variance and covariance, close to 1, over 0.9 is preferable [42]), RMSEA (the root mean square error of approximation based on population discrepancy, smaller values below 0.08 preferable [43]), and IFI (Incremental Fit Index, close to 1, over 0.9 is preferable [44]).

## Results

### Descriptive analysis

Of the 53 participants, 30 (53.6%) were homozygous for the short allele, 24 (42.9%) carried the heterogeneous genotype, and 2 (3.6%) were homozygous for the long allele. 5-HTTLPR groups did not differ significantly in regard to gender. One-way ANOVAs showed significant effects of genotype on both perceived maternal rejection and maternal emotional warmth. SL/LL carriers reported more maternal rejection, *F* (1,55) = 4.79, *p* < .05) and less emotional warmth *F* (1,55) = 4.41, *p* < .05) than SS carriers.

### Facial-Emotion Recognition Test results

**Fig 4:**
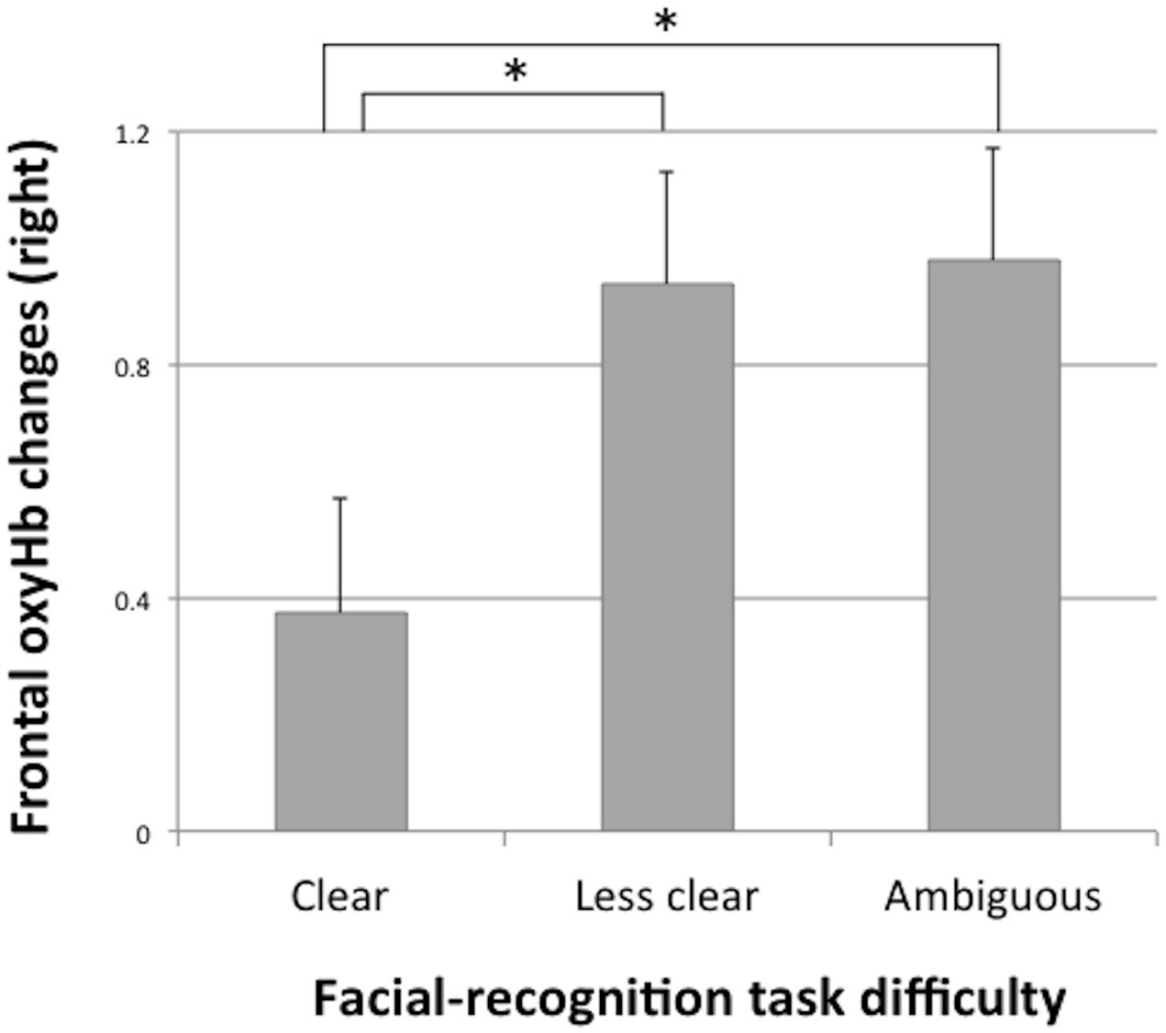
Comparison of right frontal oxy-Hb changes in the facial-recognition task difficulty. Note: * *p* < .05.

Table 1 shows mean right and left frontal activation values as a function of different facial expressions, gender, genotype, and perceived parenting style. To test whether magnitude of right frontal activation differed across facial expressions, we conducted a series of paired samples t-tests. Right frontal activation during ambiguous faces was significantly higher than that during clear (*t* (322) = 2.20, *p* < .05) or less clear expressions (*t* (322) = −10.65, *p* < .001; see Fig 4).

**Table 1:** Mean values of right and left frontal activation on different levels of facial tasks divided in gender, genotype, and perceived parenting.

Factors	All	Gender		5-HTTLPR		Rejection (father)		Rejection (mother)	
		Male	Female	SS	SL/LL	High	Low	High	Low
LoxH (Clear)	.24241	.18375	.34016	.10131	.10131	.49490	.11726	-.05119	.37039
LoxH (Less Clear)	.84297	1.0025	.57713	.84698	.84698	.77894	.88803	.67152	.91769
LoxH (Ambiguous)	.93474	1.0669	.71446	.84967	.84966	.79976	1.0141	.60224	1.0796
RoxH (Clear)	.27503	.15166	.48067	.34755	.19168	.45009	.18317	.9822	.36234
RoxH (Less Clear)	.83126	.95294	.62846	.99184	.64597	.83724	.84801	.7801	.90330
RoxH (Ambiguous)	.85452	.84678	.86743	.96856	.72294	.25674	1.1454	.7555	1.1672

LoxH, Left frontal oxygen haemoglobin; RoxH, Right frontal oxygen haemoglobin.

A Pillai’s MANOVA (2×2 factorial design) was conducted with gene and parental rejection as independent variables and NIRS recordings when rating clear, less clear and ambiguous faces as dependent variables. The analysis was conducted separately for rejection from fathers and mothers. The analysis for fathers yielded a significant main effect of paternal rejection (*p* = .015, η^2^ = .194, *power* = .790). However, there was no significant main effect on gene (*p* = .883, η^2^ = .023, *power* = .158), or an interaction (*p* = .791, η^2^ = .021, *power* = .113) between the independent variables. The analysis for mothers revealed a significant main effect of maternal rejection (*p* < .000, η^2^ = .318, *power* = .979), and an interaction between gene and maternal rejection (*p* < .005, η^2^ = .117, *power* = .744). There was no significant main effect on gene (*p* = .964, η^2^ = .014, *power* = .110).

Univariate F-tests showed significant effects of paternal and maternal rejection on RoxH, (*F* (1,50) = 7.16, *p* = .010, η^2^ = .125, *power* = .747 for fathers; *F* (1,50) = 20.94, *p* < .000, η^2^ = .295, *power* = .994 for mothers). These results suggest that individuals who perceived their father or mother as rejecting tended to have lower right frontal activation when rating ambiguous facial expressions.

**Fig 5:**
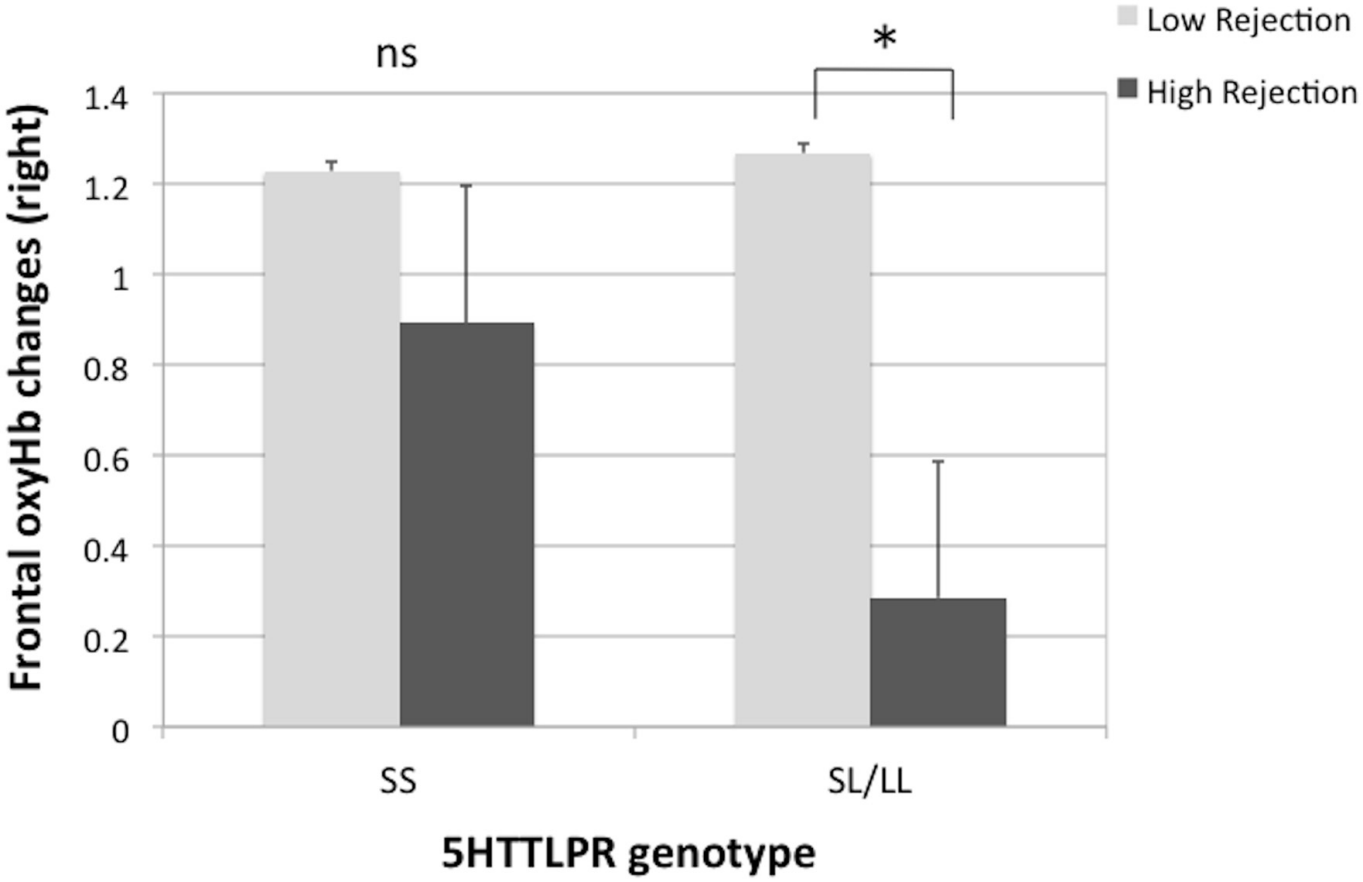
Comparisons of high/low maternal rejection in right frontal oxy-Hb changes during ambiguous facial expression task within the same genotype group. Note: * *p* < .05.

A univariate F-test showed a significant gene × maternal rejection interaction on RoxH (*F* (1,50) = 4.84, η^2^ = .162, *p* = .012, *power* = .994), suggesting that individuals who carry the SL or LL genotype and with higher perceptions of rejection from their mothers had less right frontal activation compared to the SL/LL carriers with lower levels of perceived maternal rejection (see Fig 5). The interaction was not shown for paternal rejection (*F* (1,50) = .010, *p* = .921, η^2^ = .000, *power* = .051).

### SEM Evaluation

Before testing models, intercorrelations among factors were analysed. Table 2 shows correlations between perceived parenting, right/left frontal activation and other variables. Right frontal activation during ambiguous faces was negatively associated with rejection by fathers and mothers (*r = —*.290 for fathers, *r = —*.358 for mothers, both *p* < .001), and positively associated with paternal and maternal emotional warmth (*r* = .340 for fathers, *r* = .285 for mothers, both *p* < .001).

**Table 2:** Variables assessed in the present study: Means, standard deviations, and correlations between the variables.

	Mean	SD	1	2	3	4	5	6	7	8	9	10
**1. Rejection Father**	30.15	5.08	-									
**2. Rejection Mother**	30.91	5.45	-.734**	-								
**3. Emotional Warmth Father**	47.45	9.24	-.344**	-.366**	-							
**4. Emotional Warmth Mother**	51.85	8.27	-.304**	-.469**	.778**	-						
**5. LoxH (Clear)**	.24241	.931	.030	-.158	.174	.122	-					
**6. LoxH (Less Clear)**	.84230	.842	.146	.007	.034	-.008	-	-				
**7. LoxH (Ambiguous)**	.93474	.934	-.084	-.123	.060	-.030	-	-	-			
**8. RoxH (Clear)**	.27503	.275	-.056	-.141	.167	.127	-	-	-	-		
**9. RoxH (Less Clear)**	.83126	.831	.193	.033	.072	.121	-	-	-	-	-	
**10. RoxH (Ambiguous)**	.85452	.855	**-.290** *	**-.358** **	**.340** *	**.285** *	-	-	-	-	-	-
**11.5HTTLPR(1 = SS,2 = SL/LL)**	-	-	.163	.286*	-.275*	.142	-.-.003	-.064	-.051	-.087	-.127	-.109

Note: LoxH, Left frontal oxygen haemoglobin; RoxH, Right frontal oxygen haemoglobin.

* *p* < .05,

** *p* < .001.

**Fig 6:**
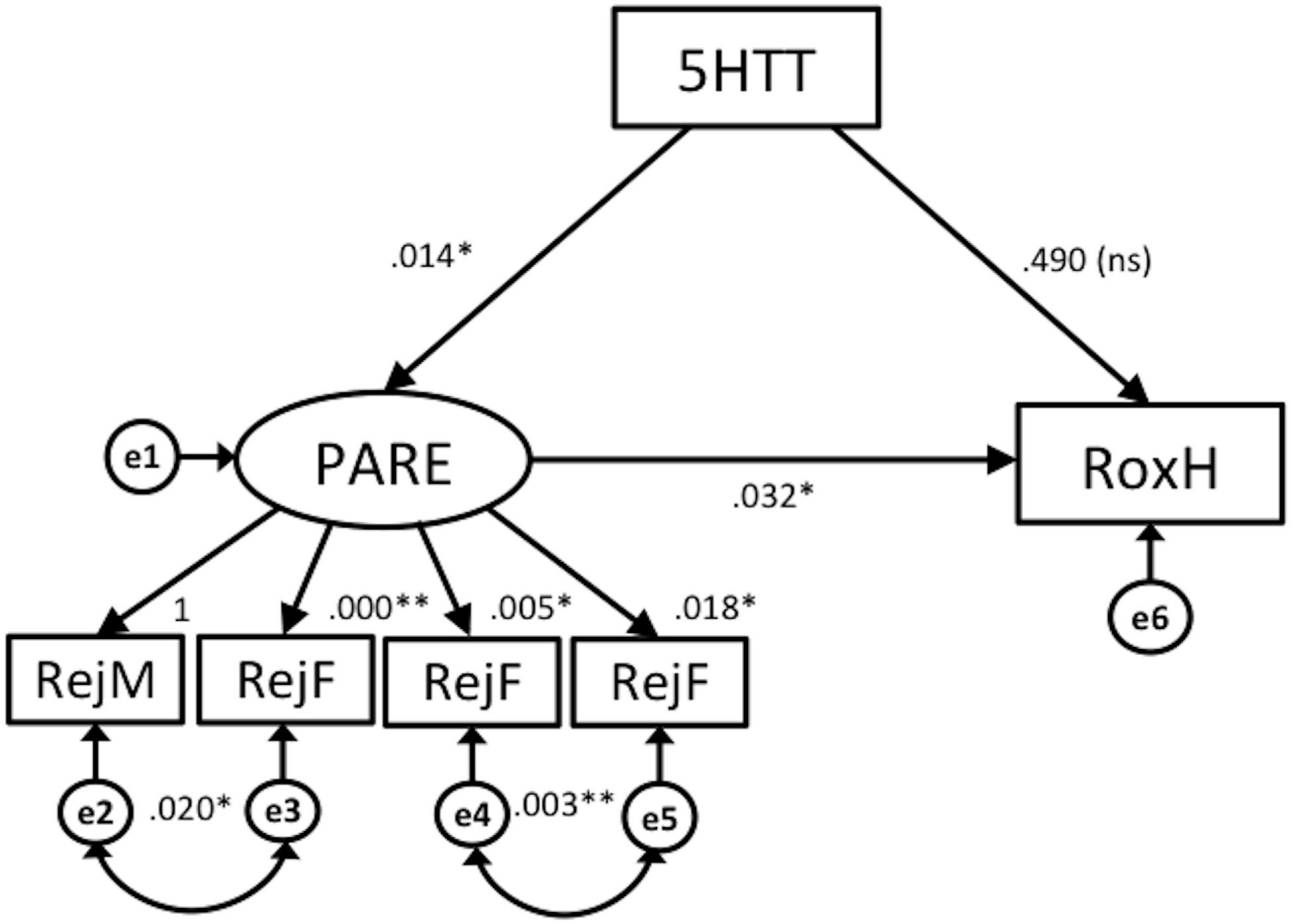
Structural Equation Model 1. Note: All paths significant (** *p* < .001 * *p* < .05) unless indicated (ns). 5HTT = 5-HTTLPR, PARE = Perceived parenting style (ReF = Paternal Rejection, ReM = Maternal Rejection, EmF = Emotional warmth from father, EmM = Emotional warmth from mother), RoxH = Right frontal oxygen hemoglobin.

Fig 6 presents the standardized SEM solution, specifying 5-HTTLPR (5HTT) as a direct determinant of perceived parental rearing (PARE) and right frontal activation during an ambiguous facial-emotion recognition task (RoxH). Following correlational analysis results, two EMBU subscales (Rejection and Emotional Warmth from fathers or mothers) were used for the model. The estimated mediational model with standardized path coefficients is presented in Table 3. The following strategy was used to test mediation. First, model 1 was explored in Fig 6. The pathways linking 5HTT, PARE, and RoxH were all significant (*p* < .05); however, the direct pathway from 5HTT to RoxH was not significant. Goodness of fit indices indicated an acceptable fit between model 1 and the data, X^2^ = 6.61 (df = 6, *p* = .359), CMIN/df = 1.10, RMSEA = .043, CFI = .94; see Table 3).

**Table 3:** Comparisons among pathways from 5-HTTLPR to perceived parenting and right frontal activation, using various measures of model fit.

	X^2^ (df)	CMIN/df	CFI	RMSEA	IFI
**Model 1**	6.60 (6)	1.10	.994	.043	.995
**Model 2**	7.08 (7)	1.01	.999	.015	.999

**Fig 7:**
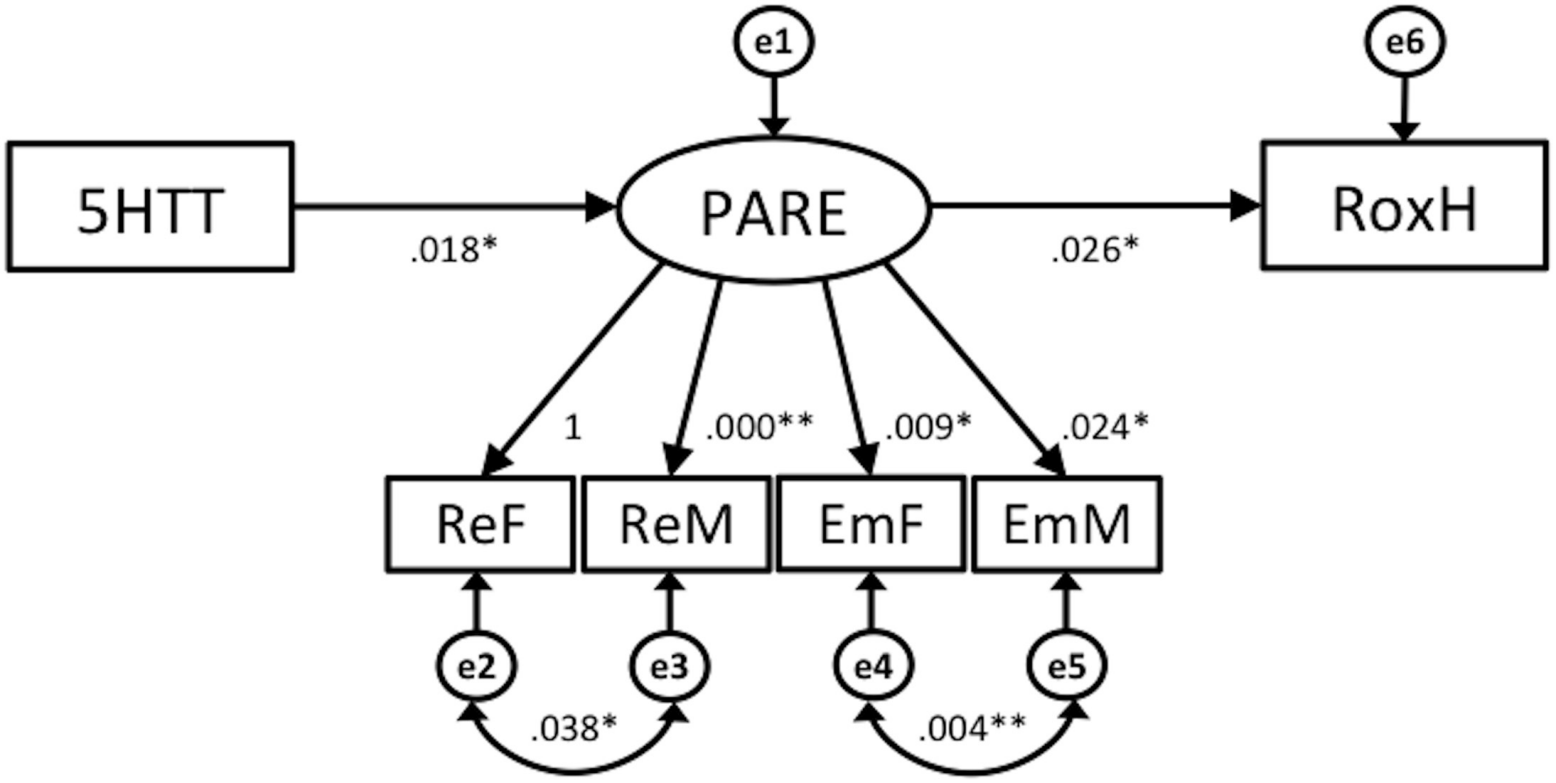
Structural Equation Model 2. Note: All paths significant (** *p* < .001 * *p* < .05) unless indicated (ns). 5HTT = 5-HTTLPR, PARE = Perceived parenting style (ReF = Paternal Rejection, ReM = Maternal Rejection, EmF = Emotional warmth from father, EmM = Emotional warmth from mother), RoxH = Right frontal oxygen hemoglobin.

The next possibility was explored in the model illustrated in Fig 7. Because the pathway between 5HTT and RoxH was not significant in model 1, model 2 was evaluated, in which this pathway is eliminated. Model 2 assessed an indirect influence of 5HTT on RoxH through PARE. Pathways linking 5HTT and PARE with RoxH were both significant (*p* < .001 and *p* < .005, respectively). The relationships between all constructs and their indicators were positive and significant (*p* < .001). Goodness of fit indices indicated an acceptable fit between model 2 and the data, X^2^ = 7.09 (df = 7, *p* = .420), CMIN/df = .772, CFI = 1.00, RMSEA = .000, IFI = .999; see Table 3).

## Discussion

The present study investigated changes in prefrontal oxy-Hb levels measured by NIRS during a facial-emotion recognition task in healthy adults, and examined interplay between as well as moderation/mediational models of the serotonin transporter gene (5-HTTLPR), perceived parenting, and their effects on cognitive/emotional processing. Correlational analysis indicated that right frontal activation when rating ambiguous expressions was negatively associated with perceived rejection by both fathers and mothers, and was positively associated with paternal and maternal emotional warmth. There was a significant main effect of maternal rejection on right frontal activation, and also a G×E interaction between maternal rejection and 5-HTTLPR on right frontal activation. Finally, SEM analysis indicated that perceived parenting style plays a mediating role in right frontal activation via the 5-HTTLPR genotype. These effects were not shown in an analysis of left frontal activation, and there was no direct effect of genotype on either right or left frontal lobe activation.

Using SEM, two models were evaluated in regard to both direct and indirect relationships between the 5-HTTLPR gene (5HTT) and right frontal activation during the facial-emotion recognition task (RoxH) via perceived parental rearing (PARE). Model 1, which features 5-HTTLPR gene as a moderator of parenting style and right frontal activation, provided a reasonable fit with the data. However, the direct path from 5HTT to RoxH was not significant. The paths featured in model 2 were all significant, and this model showed a good fit with the data.

Among SL/LL carriers, right frontal activation during the facial-emotion recognition task differed as a function of perceived parenting style. When L-allele carriers endorsed positive parenting, right frontal activation was significantly higher than for those who perceived their parents as rejecting and cold. Punitive parenting predicts error-related brain activity [45], and healthy upbringing is linked with positive development of the PFC [18,19]. Furthermore, the results showed that early parenting behaviours interact with genotype in relation to right frontal activation during a facial-emotion recognition task. This result is in line with the results of Moutsiana et al. [20], which suggested that disturbance of early caregiver relationships affects the neural regulation of emotion later in adulthood. Rating the ambiguous faces may let the participants in the uncertain situation. Moreover, it may also be possible to interpret our results such that early negative parenting influences one’s perceptions of uncertain situations, particularly among L-carriers in the present study.

We identified a main effect of perceived maternal rejection and its interaction with gene on right frontal activation during the ambiguous facial-emotion recognition task. These results may be in line with the finding of a strong influence of mothers' rearing style on individuals’ impulsive behaviours [23]. The present study showed no significant main effects of gene on right/left frontal activation during any phrase of the facial-emotion recognition task. It may be that the influence on a single gene might be small for the function of the social brain. However, when applying perceived parenting as interacting with gene, the results supported an influence of perceived parenting only among L-carriers. Despite a study showing a role of the S-allele in highlighting negative thoughts about self and environment [4], the present study showed that individuals carrying one or two L alleles perceived their mothers as more rejecting and as less emotionally warm compared to S homogenous individuals. It remains for further studies to disentangle whether this complex result can be explained on the basis of plasticity in the SS-genotype [28].

It is important to note limitations of the present study. Firstly, the EMBU questionnaire is based on retrospective self-report, and measures the subjective experiences of parental behaviour as reported by participants. We cannot say if the results of the present study are general, or specific to EMBU. For this reason, future work should analyse some other variable, for example “negative emotional traits” as an alternative to the EMBU, in order to see if the recorded effects on the NIRS are robust. Second, the effects of gender on the faces used in the recognition task are unknown. Third, using a complex study design with many variables but few participants makes the results difficult to evaluate. We were unable to perform gender comparisons in the SEM models due to the small sample size. Fourth, we could not assess the effects of parental genotype and their parenting styles. It is known that parental genotypes are related to negative parenting behaviours and of course children share the genotypes of their parents [29]. A recent study found that links between 5-HTTLPR gene of mother and child were related to child brain morphology and behavioural performance [46].

Finally, we have not investigated the functionality of the 5-HTTLPR gene in relation to the other polymorphisms and genotypes. More polymorphisms should be investigated with regard to perceived parenting and neural networks; DRD4 [47], and COMT [48] in particular. A recent study has shown that 5-HTTLPR and COMT modulated the vulnerability for anxiety disorders via different mechanisms. That is, the 5-HTTLPR influenced the quality and intensity of the fear, while COMT seemed to influence inhibition of fear in the presence of safety learning [49].

Despite limitations, the present study makes a small contribution to the mapping of a combined influence of gene and behaviour on neural system functioning. It can be speculated that neural activity engaged in facial recognition contributes to the nature of early interactions with caregiver. Perceived parenting in childhood may constitute a direct factor that affects the neural imaging of facial recognition in adulthood. 5-HTTLPR indirectly influences the neural system underlying facial recognition; however, this influence is dependent on the environment in which the individual was raised, or at least his/her perception of said environment. Furthermore, early childhood experiences may influence neural activity in uncertain situations, in certain genotypes. These should be clarified in the further studies with a sufficient sample size and use of psychophysiologically well-designed experimental tasks. Such an approach would provide further opportunities for further studies of clinical samples as well as children, in order to clarify links and characteristic G×E interaction effects on our cognitive and emotional processes.
